# Renal Anomalies Associated with Ectopic Neurohypophysis

**DOI:** 10.4274/jcrpe.v3i2.12

**Published:** 2011-06-08

**Authors:** Samim Özen, Damla Gökşen Şişmek, Asan Önder, Şükran Darcan

**Affiliations:** 1 Pediatric Endocrinology Unit, Mersin Children Hospital, Toroslar, Mersin, Turkey; 2 2Department of Pediatric Endocrinology and Metabolism, Ege University School of Medicine, Izmir, Turkey; 3 3Department of Pediatrics, Ege University School of Medicine, Izmir, Turkey

**Keywords:** Ectopic neurohypophysis, renal anomalies, pituitary hormone deficiency

## Abstract

**Objective: ** Although the etiology of ectopic neurohypophysis that leads to pituitary hormone deficiencies is not yet clearly understood, birth trauma or genetic factors have been considered responsible. Concurrent cranial and extracranial congenital anomalies have been reported in such cases. The aim of the present study was to investigate the frequency of renal anomalies in nonsyndromic cases with ectopic neurohypophysis.

**Methods:** We retrospectively evaluated the medical records of 20 patients with ectopic neurohypophysis who were followed up between January 1990 and December 2007 in a tertiary University Hospital.

**Results:** Renal anomalies were identified in three (15%) cases including unilateral renal agenesis in one case, renal hypoplasia in one case, and double collecting system and unilateral renal agenesis in one case.

**Conclusions:** In the present study, the increased frequency of renal anomalies in cases of ectopic neurohypophysis was highlighted, and it was emphasized that there might be common genetic factors that lead to such associations.

**Conflict of interest:**None declared.

## INTRODUCTION

Ectopic neurohypophysis is frequently associated with anterior pituitary hormone deficiencies. Isolated growth hormone deficiency (GHD) or multiple pituitary hormone deficiency (MPHD), in which GHD is associated with one or more other anterior pituitary hormone deficiencies, may develop in these cases. Although the etiology is unclear, traumatic birth, breech delivery and genetic factors have all been considered as possible factors (1,2). Some of the genetic factors include LHX4, HESX1, GLI2, GH-1, GH releasing hormone (GHRH) receptor, POU1F1, PROP-1and SOX3 mutations and have been shown to be important in the development of the pituitary gland. However, studies have failed to implicate a genetic factor in 95% of cases of ectopic neurohypophysis associated with GHD (1,2,3,4,5). In the present study, nonsyndromic cases with ectopic neurohypophysis were evaluated with respect to renal developmental anomalies, while the etiology of GHD was being investigated.

## MATERIALS AND METHODS

**Patients**

Two-hundred and thirteen cases followed between January 1990 and December 2007 at the Pediatric Endocrinology Department of Ege University Faculty of Medicine with the diagnosis of GHD were included in this study. The medical records of the patients were retrospectively evaluated. Of the total group, 20 (9.3%) cases (7 girls and 13 boys), in whom ectopic neurohypophysis was detected via magnetic resonance imaging (MRI), were included in this study. We analyzed the data regarding chronologic age at the time of diagnosis, standard deviation scores (SDSs) for height and body mass index (BMI) at the time of diagnosis, history of parental consanguinity, difficult birth history, family history of short stature, presence of other pituitary hormone deficiencies or additional anomalies other than ectopic neurohypophysis and presence of renal anomalies. Sagittal and coronal 3-mm-thick slices of the pituitary area were obtained via MRI. All MRI scans were assessed by pediatric neuroradiologists who were not blinded to the clinical information.

Two standard GH stimulation tests were performed with insulin and levodopa (L-DOPA). In one neonate, plasma GH levels were measured during spontaneous hypoglycemia. A peak value of less than 10 ng/mL in two tests was regarded as confirmatory for the diagnosis of GHD. Adrenocorticotropic hormone (ACTH) deficiency was diagnosed based on a cortisol level of less than 5 μg/dL and a peak cortisol level of less than 25 μg/dL on ACTH test. The diagnosis of thyroid-stimulating hormone (TSH) deficiency was based on a free thyroxine (fT4) level of less than 0.8 ng/dL in combination with a normal TSH level. Moreover, patients with TSH levels below the normal range were also diagnosed as having TSH deficiency. Thyrotropin-releasing hormone (TRH) stimulation test (7 μg/kg) was also used in the diagnosis of TSH deficiency. Hypogonadotropic hypogonadism was suspected when both luteinizing hormone (LH) and follicle-stimulating hormone (FSH) responses to LH-releasing hormone (LHRH) stimulation test (100 μg/m2) were flat in pubertal patients. Basal and stimulated levels of cortisol, ACTH, TSH, prolactin (PRL), FSH, and LH were measured at sequential time points (15, 30, 60 and 90 minute after stimulation). Each hormone was measured by immunochemiluminescent assay and radioluminescence. Renal anomalies were examined by experienced radiologists via ultrasonography (US). In case of pathological findings on US, the presence of renal anomalies was confirmed via renal scintigraphy and intravenous pyelography (IVP).

This study was conducted according to the principles of the Declaration of Helsinki. 

## RESULTS

The mean age of the patients at the time of diagnosis was 11.3±4.7 years (range, 0.2-18.1 years). The mean SDS for height at the time of diagnosis was -4.2±1.5 and the mean SDS for BMI at the time of diagnosis was -0.2±1.2. Five (20%) of the cases had a family history of short stature and two (10%) had a history of parental consanguinity. Two cases had a history of difficult birth. 

Endocrinological evaluation of the patients, in whom ectopic neurohypophysis was detected on MRI, revealed that five (20%) cases had isolated GHD and 15 (80%) had MPHD. GHD was followed by TSH deficiency (13/20; 65%), FSH-LH deficiency (9/20; 45%) and ACTH deficiency (4/20; 20%), in decreasing order.

MRI scans revealed stalk formation in three cases. One of these cases had isolated GHD and two had MPHD. MPHD was determined in all of the 17 cases without stalk formation on MRI.

Examination of the patients for renal anomalies revealed renal anomalies in three (15%) cases: unilateral renal agenesis in one case, renal hypoplasia in one case, and double collecting system and unilateral renal agenesis in one case. All of the cases with renal anomalies had also MPHD.

Two patients had testicular hypoplasia, one case had chromosomal abnormality [46, XX/47, XX + mar (6) karyotype], and one case had Fanconi anemia. Table 1 shows the clinical characteristics, endocrinological deficiencies of the patients as well as the presence of renal and additional anomalies. None of the patients had a family history of renal anomalies. 

**Table 1:**
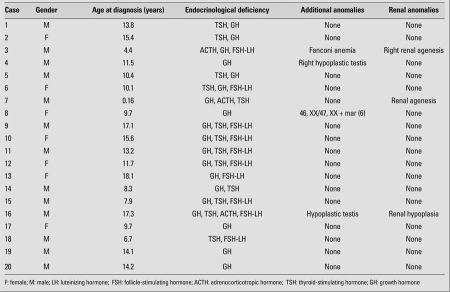
Clinical features, endocrinological deficiencies, renal and other additional anomalies of the patients

## References

[1] Rosenbloom AL, Connor EL (2007). Hypopituitarism and other disorders of the growth hormone- insulin like growth factor-1 axis. In: Lifshitz F (ed). Pediatric Endocrinology..

[2] Rosenfeld RG, Cohen P (2008). Disorders of growth hormone/insulin-like growth factor secretion and action. In: Sperling M (ed)..

[3] di Iorgi N, Secco A, Napoli F, Calandra E, Rossi A, Maghnie M (2009). Developmental abnormalities of the posterior pituitary gland.. Endocr Dev.

[4] Mehta A, Dattani MT (2008). Developmental disorders of the hypothalamus and pituitary gland associated with congenital hypopituitarism.. Best Pract Res Clin Endocrinol Metab.

[5] 5.	Topaloglu A (2009). Genetics of growth hormone deficiency.. J Clin Res Ped Endo.

[6] Maghnie M, Larizza D, Triulzi F, Sampaolo P, Scotti G, Severi F (1991). Hypopituitarism and stalk agenesis: a congenital syndrome worsened by breech delivery?. Horm Res.

[7] Simon D, Hadjiathanasiou C, Garel C, Czernichow P, Léger J (2006). Phenotypic variability in children with growth hormone deficiency associated with posterior pituitary ectopia.. Clin Endocrinol (Oxf).

[8] Parano E, Trifiletti RR, Barone R, Pavone V, Pavone P (2000). Arthrogryposis multiplex congenita and pituitary ectopia. A case report.. Neuropediatrics.

[9] Dupuis-Girod S, Gluckman E, Souberbielle JC, Brauner R (2001). Growth hormone deficiency caused by pituitary stalk interruption in Fanconi's anemia.. J Pediatr.

[10] Halâsz Z, Bertalan R, Toke J, Patócs A, Tóth M, Fekete G, Glâz E, Râcz K (2008). Laterality disturbance and hypopituitarism. A case report of co-existing situs inversus totalis and combined pituitary hormone deficiency.. J Endocrinol Invest.

[11] Mullis PE (2000). Transcription factors in pituitary gland development and their clinical impact on phenotype.. Horm Res.

[12] Machinis K, Pantel J, Netchine I, Léger J, Camand OJ, Sobrier ML, Dastot-Le Moal F, Duquesnoy P, Abitbol M, Czernichow P, Amselem S (2001). Syndromic short stature in patients with a germline mutation in the LIM homeobox LHX4.. Am J Hum Genet.

[13] Thomas PQ, Dattani MT, Brickman JM, McNay D, Warne G, Zacharin M, Cameron F, Hurst J, Woods K, Dunger D, Stanhope R, Forrest S, Robinson IC, Beddington RS (2001). Heterozygous HESX1 mutations associated with isolated congenital pituitary hypoplasia and septo-optic dysplasia.. Hum Mol Genet.

[14] Carvalho LR, Woods KS, Mendonca BB, Marcal N, Zamparini AL, Stifani S, Brickman JM, Arnhold IJ, Dattani MT (2003). A homozygous mutation in HESX1 is associated with evolving hypopituitarism due to impaired repressor-corepressor interaction.. J Clin Invest.

[15] Roessler E, Du YZ, Mullor JL, Casas E, Allen WP, Gillessen-Kaesbach G, Roeder ER, Ming JE, Ruiz i Altaba A, Muenke M (2003). Loss-of-function mutations in the human GLI2 gene are associated with pituitary anomalies and holoprosencephaly-like features.. Proc Natl Acad Sci U S A.

[16] Sanna-Cherchi S, Caridi G, Weng PL, Scolari F, Perfumo F, Gharavi AG, Ghiggeri GM (2007). Genetic approaches to human renal agenesis/hypoplasia and dysplasia.. Pediatr Nephrol.

[17] Shawlot W, Behringer RR (1995). Requirement for Lim1 in head-organizer function.. Nature.

[18] Kobayashi A, Kwan KM, Carroll TJ, McMahon AP, Mendelsohn CL, Behringer RR (2005). Distinct and sequential tissue-specific activities of the LIM-class homeobox gene Lim1 for tubular morphogenesis during kidney development.. Development.

[19] Nishinakamura R, Matsumoto Y, Nakao K, Nakamura K, Sato A, Copeland NG, Gilbert DJ, Jenkins NA, Scully S, Lacey DL, Katsuki M, Asashima M, Yokota T (2001). Murine homolog of SALL1 is essential for ureteric bud invasion in kidney development.. Development.

[20] Poladia DP, Kish K, Kutay B, Hains D, Kegg H, Zhao H, Bates CM (2006). Role of fibroblast growth factor receptors 1 and 2 in the metanephric mesenchyme.. Dev Biol.

[21] Xu PX, Zheng W, Huang L, Maire P, Laclef C, Silvius D (2003). Six1 is required for the early organogenesis of mammalian kidney.. Development.

[22] Self M, Lagutin OV, Bowling B, Hendrix J, Cai Y, Dressler GR, Oliver G (2006). Six2 is required for suppression of nephrogenesis and progenitor renewal in the developing kidney.. EMBO J.

[23] Johnson KR, Cook SA, Erway LC, Matthews AN, Sanford LP, Paradies NE, Friedman RA (1999). Inner ear and kidney anomalies caused by IAP insertion in an intron of the Eya1 gene in a mouse model of BOR syndrome.. Hum Mol Genet.

[24] Johnston JJ, Sapp JC, Turner JT, Amor D, Aftimos S, Aleck KA, Bocian M, Bodurtha JN, Cox GF, Curry CJ, Day R, Donnai D, Field M, Fujiwara I, Gabbett M, Gal M, Graham JM, Hedera P, Hennekam RC, Hersh JH, Hopkin RJ, Kayserili H, Kidd AM, Kimonis V, Lin AE, Lynch SA, Maisenbacher M, Mansour S, McGaughran J, Mehta L, Murphy H, Raygada M, Robin NH, Rope AF, Rosenbaum KN, Schaefer GB, Shealy A, Smith W, Soller M, Sommer A, Stalker HJ, Steiner B, Stephan MJ, Tilstra D, Tomkins S, Trapane P, Tsai AC, Van Allen MI, Vasudevan PC, Zabel B, Zunich J, Black GC, Biesecker LG (2010). Molecular analysis expands the spectrum of phenotypes associated with GLI3 mutations.. Hum Mutat.

[25] Kirk JM, Grant DB, Besser GM, Shalet S, Quinton R, Smith CS, White M, Edwards O, Bouloux PM (1994). Unilateral renal aplasia in X-linked Kallmann's syndrome.. Clin Genet.

[26] Wegenke JD, Uehling DT, Wear JB Jr, Gordon ES, Bargman JG, Deacon JS, Herrmann JP, Opitz JM (1975). Familial Kallmann syndrome with unilateral renal aplasia.. Clin Genet.

[27] Hermanussen M, Sippell WG (1985). Heterogeneity of Kallmann's syndrome.. Clin Genet.

